# Serum Exosomal Proteins F9 and TSP-1 as Potential Diagnostic Biomarkers for Newly Diagnosed Epilepsy

**DOI:** 10.3389/fnins.2020.00737

**Published:** 2020-08-04

**Authors:** Zijun Lin, Yixue Gu, Ruijiao Zhou, Meiling Wang, Yi Guo, Yuanyuan Chen, Junhong Ma, Fei Xiao, Xuefeng Wang, Xin Tian

**Affiliations:** ^1^Department of Neurology, The First Affiliated Hospital of Chongqing Medical University, Chongqing Key Laboratory of Neurology, Chongqing, China; ^2^Center of Epilepsy, Beijing Institute for Brain Disorders, Beijing, China

**Keywords:** epilepsy, exosome, TMT, biomarkers, TSP-1, F9

## Abstract

Epilepsy is one of the most common chronic neurological diseases in the world, with a high incidence, a high risk of sudden unexplained death, and diagnostic challenges. Exosomes are nanosized extracellular vesicles that are released into physical environments and carry a variety of biological information. Moreover, exosomes can also be synthesized and released from brain cells, passing through the blood-brain barrier, and can be detected in peripheral blood or cerebrospinal fluid. Our study using the tandem mass tag (TMT) approach showed that a total of 76 proteins were differentially expressed in serum exosomes between epilepsy patients and healthy controls, with 6 proteins increasing and 70 proteins decreasing. Analysis of large clinical samples and two mouse models of chronic epilepsy indicated that two significantly differentially expressed serum exosomal proteins, coagulation factor IX (F9) and thrombospondin-1 (TSP-1), represent promising biomarkers for the diagnosis of epilepsy, with area under the curve (AUC) values of up to 0.7776 (95% CI, 0.7306–0.8246) and 0.8534 (95% CI, 0.8152–0.8916), respectively. This is the first study of exosomal proteins in epilepsy, and it suggests that exosomes are promising new tools for the diagnosis of epilepsy.

## Introduction

Epilepsy is one of the most common chronic neurological diseases in the world, and it affects people of all ages, without geographic, social or ethnic boundaries. The incidence of epilepsy is substantial. Worldwide, 50 million people suffer from epilepsy; moreover, an estimated 2.4 million people are diagnosed with epilepsy each year, and the prevalence of epilepsy in developed and developing countries is 4–10 per 1,000 people and 14 to 57 per 1,000 people, respectively (Burneo et al., 2005; Carpio and Hauser, 2009). The risk of premature death in epilepsy patients is significantly higher than that in the general population (Nevalainen et al., 2014). There are 180,000 epilepsy-related deaths worldwide each year (Lozano et al., 2012). Sudden unexplained death in epilepsy (SUDEP), which will soon become the second most common cause of decline in adult life years after stroke, accounts for an estimated 1.16 deaths per 1,000 epilepsy patients (Thurman et al., 2014). Drugs are the main treatment for epilepsy. However, there is literature showing that although early antiepileptic drug (AED) treatment effectively reduces the risk of recurrence of seizures in the short term, seizures remain uncontrolled in approximately 30% of patients, and side effects often occur (Fiest et al., 2017).

The diagnosis of epilepsy is challenging, with up to 25% of patients considered to have a false-positive diagnosis (Leach et al., 2005). The diagnosis of epilepsy mainly depends on clinical examination and medical history (Moshé et al., 2015). However, even in a professional epilepsy monitoring unit that can obtain continuous video electroencephalogram (EEG) recording, the diagnostic value of EEG is still limited (Engel et al., 2013). Epilepsy has a complex classification and different clinical manifestations that pose great challenges for diagnosis. Therefore, molecular biomarkers that provide rapid, inexpensive, and non-invasive support for the diagnosis of epilepsy are of great value (Pitkänen et al., 2016).

Exosomes have received increasing attention in the medical community due to their outstanding transmission ability and excellent stability. Exosomes are endothelium-derived extracellular vesicles (EVs) of approximately 30–150 nm in size that are widely present in bodily fluids and rich in nucleic acids, proteins, cholesterol, etc. The exosomes released into the peripheral circulation by healthy subjects and patients contain different proteins, which can be measured as biomarkers (Clayton et al., 2003). Based on the structural and biological characteristics of exosomes, we speculate that exosomal proteins can be used as biomarkers of epilepsy. Moreover, exosomes can also be used to study the mechanism of epilepsy.

## Materials and Methods

### Patient Recruitment and Blood Collection

In this study, 200 epilepsy patients and 200 healthy controls were recruited at the First Affiliated Hospital of Chongqing Medical University (Table 1). The 200 epilepsy patients were newly diagnosed according to the guidelines of the International League Against Epilepsy (ILAE) (Berg et al., 2010; Fisher et al., 2014) and had never used any treatments or taken any medication to control their seizures. Blood samples were collected by venipuncture into a blood collection tube without any anticoagulant.

**TABLE 1:** Demographic and clinical data.

**Characteristics**	**Age (range)**	**Sex (Male**	**Female)**	**Number**
Epilepsy	32.8 years (18–53)	104	96	200
Health control	35.5 years (22–62)	92	108	200

### Human Brain Tissues

Four patient samples (two males and two females) originally acquired at the First Affiliated Hospital of Chongqing Medical University were selected randomly from our laboratory’s established brain tissue bank as previously described (Zhang et al., 2016). The selection was made from patients who had been diagnosed with temporal lobe epilepsy (TLE) according to the criteria established by the ILAE. The control cases included four patients (two males and two females) treated for traumatic brain injury (TBI) at the First Affiliated Hospital of Chongqing Medical University. TLE is one of the major causes of focal epilepsy and is characterized clinically by the progressive development of spontaneous recurrent seizures originating in the temporal lobe; approximately 70–80% of patients with TLE develop drug-refractory epilepsy (Ramey et al., 2013). Clinical data for all of the patients are shown in Table 2. There were no significant differences in age or sex distribution between the TLE patients and the controls (*n* = 4 in each group, *P* > 0.05).

**TABLE 2:** The clinical characterization of TLE patients and controls.

**Patient**	**Age (years)**	**Duration (years)**	**Preoperative AED consumption**	**Side of resected temporal lobe**	**Pathological diagnosis**
**TLE patients**					
E1	20–25	3	OXC/LEV/PB	L	NL, G
E2	10–15	4	LTG/TPM/PB	R	NL, G
E3	35–40	8	CBZ/VPA/LEV/PB	R	NL, G
E4	65–70	33	PHT/TPM/PB	L	G
**Patient**	**Age (years)**	**Disease diagnosis**	**Preoperative AED consumption**	**Side of resected temporal lobe**	**Pathological diagnosis**
**Control patients**					
C1	15–20	Brain trauma	None	L	Normal
C2	35–40	Brain trauma	None	R	Normal
C3	25–30	Brain trauma	None	L	Normal
C4	35–40	Brain trauma	None	R	Normal

*TLE, Temporal Lobe Epilepsy; E, epilepsy; AEDs, antiepileptic drugs; VPA, valproic acid; LTG, lamotrigine; LEV, levetiracetam; CBZ, carbamazepine; TPM, topiramate; PB, phenobarbital; PHT, phenytoin; OXC, oxcarbazepine; L, left; R, right; NL, neuron loss; G, gliosis; C, control.*

### Standard Protocol for Exosome Isolation

Exosome enrichment from brain tissue: Exosomes were isolated and purified according to an isolation protocol established by Vella et al. (2017). Frozen brain tissues were sliced lengthways on ice using a razor blade. The partially frozen cut tissue sections were weighed and transferred to a 50 ml tube containing 75 U/ml collagenase type 3 in Hibernate-E. The tissue was then incubated in a shaking water bath at 37°C for a total of 20 min. The dissociated tissue was centrifuged at 300 × *g* for 5 min at 4°C, and the supernatant was transferred to a fresh tube and centrifuged at 2000 × *g* for 10 min at 4°C. The supernatant was then centrifuged at 10,000 × *g* for 30 min at 4°C. The supernatant was overlaid on a triple sucrose cushion and centrifuged for 3 h at 180,000 × *g* (average) at 4°C. Each fraction was diluted with ice-cold phosphate-buffered saline (PBS) and centrifuged at 100,000 × *g* at 4°C for 1 h to pellet the vesicles. Following centrifugation, the supernatant was discarded, and the pellets were collected.

Exosome enrichment from serum: Serum was first collected by centrifugation at 2000 × *g* for 20 min after blood coagulation. Exosomes were isolated and purified according to an isolation protocol established by Théry et al. (2006). Serum was diluted with an equal volume of PBS, transferred to 50-ml tubes, and centrifuged for 30 min at 2,000 × *g*, 4°C. The supernatant was transferred to ultracentrifuge tubes and centrifuged for 45 min at 12,000 × *g* and 4°C. The supernatant was transferred to ultracentrifuge tubes and centrifuged for 2 h at 110,000 × *g* and 4°C. The pellet was resuspended in PBS, pooled in new tubes, filtered through a 0.22-μm filter, collected in a fresh ultracentrifuge tube, and centrifuged for 70 min at 110,000 × *g* at 4°C. The supernatant was poured off, and the pellet was resuspended in PBS and centrifuged for 70 min at 110,000 × *g* and 4°C. The supernatant was poured off, and the pellet was resuspended in PBS.

### Transmission Electron Microscopy and Nanoparticle-Tracking Analysis

For transmission electron microscopy (TEM), the isolated exosomes were fixed in 2% paraformaldehyde, followed by image acquisition on a transmission electron microscope (HITACHI HT7700) operated at 70 kV.

Exosome size and count were determined by a nanoparticle characterization system. Nanoparticle-tracking analysis (NTA) was performed using a ZetaView PMX110 analyzer (Particle Metrix, Meerbusch, Germany) and its corresponding software (ZetaView 8.02.28). Each sample was analyzed three times, and each analysis consisted of three recordings of 40 s each. As the size distributions were highly skewed, the modes were reported for particle size.

### Western Blot Analysis

Western blot analysis was performed according to published protocols (Li et al., 2016). The following primary antibodies were used in this study: rabbit anti-CD9 (1:1000, Abcam, ab92726), rabbit anti-CD63 (1:1000, Abcam, ab134045), rabbit anti-TSG101 (1:1000, Abcam, ab133586), rabbit anti-F9 (1;500, Proteintech, 21481-1-AP), rabbit anti-TSP-1 (Proteintech, 18304-1-AP), rabbit anti-amyloid-beta precursor protein (APP) (Proteintech, 25524-1-AP), and rabbit anti-GAPDH (1:3000, Thermo Scientific, PA1–987).

### Tandem Mass Tag Proteomics Analysis

The exosomal samples were removed from the frozen state, and urea and protease inhibitor were added. The samples were then lysed on ice for 30 min and centrifuged to obtain the supernatant. A bicinchoninic acid (BCA) protein concentration determination kit (Beyotime) was used to determine the protein concentration. A sodium dodecyl sulfate-polyacrylamide gel electrophoresis (SDS-PAGE) kit (Beyotime) was used to analyze the protein samples and evaluate the quality of the samples. The protein samples were subjected to reductive alkylation and enzymolysis. Protein samples were treated with tris(2-carboxyethyl)phosphine (Thermo Fisher Scientific) and reacted at 37°C for 60 min. Then, iodoacetamide I (Sigma) was added and reacted at room temperature in the dark for 40 min. Prechilled acetone (acetone:sample volume ratio = 6:1) was added to each sample. The samples were reacted at −20°C for 4 h and centrifuged at 10,000 *g* for 20 min, and the precipitate was collected. Triethylammonium bicarbonate buffer (TEAB, Sigma) was added to fully dissolve the sample, and trypsin (Promega) was added at a mass ratio of 1:50 (enzyme:protein) at 37°C overnight.

Tandem Mass Tag (TMT) reagent (Thermo Fisher Cat. No. 90111) was added to the peptide and reacted at room temperature for 2 h. Then, hydroxylamine was added and reacted at room temperature for 15 min. Reverse-phase liquid chromatography (RPLC) one-dimensional separation: Peptide samples were reconstituted with high-performance liquid chromatography (UPLC) loading buffer, and high-pH liquid phase separation was performed on a reverse-phase C18 column. Column information: ACQUITY UPLC BEH C18 Column 1.7 μm, 2.1 mm X 150 mm (Waters, United States); chromatographic instrument: Waters ACQUITY UPLC; phase A: 2% acetonitrile (ACN) and phase B: 80% can; UV detection wavelength: 214 nm; flow rate: 200 μl/min; gradient: 76 min. A total of 20 fractions were collected according to the peak shape and time, combined into 10 fractions, and then concentrated by vacuum centrifugation. Two-dimensional analysis was performed by liquid tandem mass spectrometry (Easy-nLC 1200 combined with a Q-Exactive mass spectrometer). Software: Thermo Xcalibur 4.0 (Thermo, United States); reversed-phase column information: C18 column (75 μm × 25 cm, Thermo, United States); chromatographic instrument: EASY-nLC 1200; mass spectrometer: Q-Exactive (Thermo, United States); separation time: 90 min; phase A: 2% ACN with 0.1% formic acid and phase B: 80% ACN with 0.1% formic acid; flow rate: 300 nL/min; MS scan range (m/z) 350–1300; top 20 precursor ions underwent secondary fragmentation; dynamic exclusion time was 18 s; mass spectral resolution was 70 and 35 K, respectively. The Sequest or Mascot module in Proteome Discoverer was used to search the database. Statistical and bioinformatics analyses were performed on the results of the database search.

### Enzyme-Linked Immunosorbent Assay

#### Typical Operating Procedure

Reagents and standards were prepared. One hundred microliters of sample or standard was added to each well and reacted at 37°C for 90 min without washing. Then, 100 μl of concentrated biotinylated detection Ab was added to each well, incubated at 37°C for 60 min and washed three times. Then, 100 μl avidin-biotin-peroxidase complex was added to each well and reacted at 37°C for 30 min. The wells were then washed five times. Tetramethylbenzidine (TMB) was added and reacted at 37°C in the dark for 15 min. TMB stop solution was added, and the samples were read. The optical density (OD value) of each well was measured at a wavelength of 450 nm. Protein extracts from serum exosomes were further analyzed using Human F9 (Elabscience Biotechnology, Wuhan; Catalog #E-EL-H0760c) and Human TSP-1 (Boster Biological Technology, Wuhan, China; Catalog #EK0899) enzyme-linked immunosorbent assay (ELISA) kits. The assay was performed according to the manufacturer’s instructions.

### Experimental Animals

For the epilepsy models, specific pathogen–free C57BL/6 male mice (20–25 g) were housed in the Experimental Animal Center of Chongqing Medical University. The mice were housed under standard conditions (room temperature, 23 ± 1°C; illumination, 12-h light/12-h dark cycle; *ad libitum* access to food and water).

### Mouse Models of Chronic Epilepsy

C57BL/6 mice were subjected to two classic chronic epilepsy models. Pentylenetetrazole (PTZ)-kindling epilepsy model: According to the previously described method (Zhu et al., 2017), PTZ (35 mg/kg) was administered to C57BL/6 mice (*n* = 25) by intraperitoneal (i.p.) injection for 30 days. We observed and recorded the evoked behavioral seizures in the mice for 30 min on the basis of the standard Racine scale (Phelan et al., 2015). Mice that showed at least three consecutive level 4 or 5 epileptic seizures after receiving PTZ injections were considered completely kindled, and we classified those mice as the epilepsy group. The mice that did not meet the above conditions were regarded as the control group. Kainic acid-induced (KA-induced) epilepsy model: According to the previously described method (Sada et al., 2015), C57BL/6 mice (*n* = 25) were deeply anesthetized and unilaterally injected with 1.0 nmol of KA (Sigma-Aldrich Co., St. Louis, MO, United States) in 50 nl of saline in the right hippocampus. Stereotaxic injections into the dorsal region of the CA1 area were performed. For 4 weeks, beginning 1 day after status epilepticus induction, the chronic epilepsy model was confirmed by checking for at least one behavioral spontaneous recurrent seizure (SRS) with motor manifestations. Only mice with observed SRSs during the chronic phase were included in the epilepsy group, and mice that did not exhibit SRSs were used as controls.

### Statistical Analysis

All statistical analyses were conducted using SPSS 19.0 statistical software. According to whether the samples exhibited a normal distribution and an equal variance, the experimental results were statistically assessed using parametric or non-parametric tests. For independent samples, independent Student’s *t*-test or the Mann–Whitney *U* test was performed to compare two groups. The χ2 test was applied to explore the gender differences between the groups of epilepsy patients and controls. Graphs were prepared with GraphPad Prism 7.0 software. The data from this study are expressed as the mean ± SEM. *P* < 0.05 was considered statistically significant and was indicated as ^∗^*P* < 0.05, ^∗∗^*P* < 0.01, or ^****^P < 0.0001 in the summary graphs.

## Results

### Identification of Exosomes Extracted From Serum and the Brain

**FIGURE 1:**
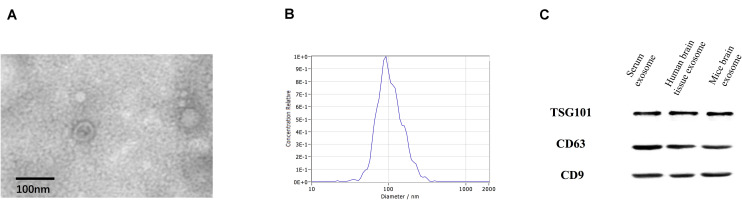
Identification of exosomes. Characterization of exosome-like vesicles by **(A)** transmission electron microscopy, **(B)** nanoparticle-tracking analysis (140 ± 19.1 nm) and **(C)** Western blotting. Presence of the exosomal markers TSG101, CD63, and CD9.

Transmission electron microscopy analysis revealed the presence of a round, cup-shaped, double-membrane-bound, vesicle-like structure (Figure 1A). Analysis by NTA: the population of spherical nanoparticles moving under Brownian motion had a peak abundance ranging between 30 and 150 nm (Figure 1B). Western blot analysis confirmed the exosome markers TSG101, CD63, and CD9, indicating that exosomes were successfully extracted by ultracentrifugation (Figure 1C).

### Proteomic Analysis Reveals That Serum Exosomal Proteins Are Differentially Expressed in Patients With Epilepsy Compared With Healthy Controls

**FIGURE 2:**
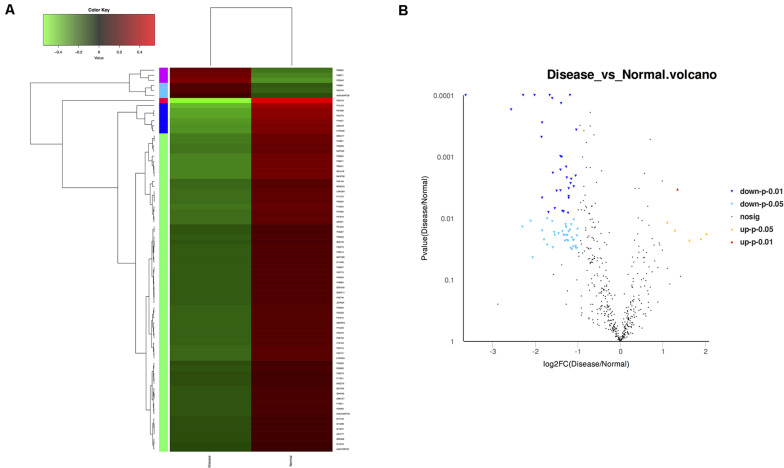
Microarray assay of exosomal proteins differentially expressed in the serum from epilepsy patients and healthy controls. **(A)** Heat map showing the differential expression of exosomal proteins in epilepsy patients compared to healthy controls. Each column represents an individual sample, and each row represents a single protein. The expression level of each protein in a single sample is depicted according to the color scale. Red represents high expression, whereas green represents low expression. **(B)** The volcano plot shows the relation between the logarithm of the *P*-values on the *y*-axis and the log fold change between epilepsy patients and healthy controls on the *x*-axis. Each point in the figure represents a specific protein. Yellow dots indicate proteins that are significantly upregulated at *P* < 0.05, red dots indicate proteins that are significantly upregulated at *P* < 0.01, light blue dots indicate proteins that are significantly downregulated at *P* < 0.05, blue dots indicate proteins that are significantly downregulated at *P* < 0.01, and black dots represent non-significantly differentially expressed proteins.

High-throughput sequencing of serum exosomal proteins in five epilepsy patients and five healthy controls was performed using proteomics. The results identified a total of 500 proteins, and 76 exosomal proteins were differentially expressed (Table 3). Compared with those in the healthy control group, the expression levels of six proteins were significantly increased and those of 70 proteins were significantly reduced in the epilepsy group (FC > 2; *P* < 0.05). The heat map and the scatter plot of proteins differentially expressed between epilepsy patients and healthy controls are shown in Figure 2. Information on the 76 differentially expressed proteins is listed in Table 3 (with fold changes and *P*-values).

**TABLE 3:** Seventy-six proteins were differentially expressed between epilepsy patients and healthy controls.

**Protein name**	**FC**	**Style**	**P**	**No.**	**Protein name**	**FC**	**Style**	**P**	**No.**
Complement C1s subcomponent	−1.851121	Down	0.000481	1	Tetraspanin	−1.454860	Down	0.017822	39
Thrombospondin-1	−2.291547	Down	0.013858	2	Integrin-linked protein kinase	−1.148552	Down	0.012722	40
Complement C1r subcomponent	−1.837532	Down	0.000280	3	Choline transporter-like protein 1	−1.339040	Down	0.007811	41
Multimerin-1	−1.053039	Down	0.028369	4	Solute carrier family 2, facilitated glucose transporter member 14	−1.227093	Down	0.023320	42
Hemoglobin subunit beta	1.884644	Up	0.021696	5	Annexin A3	−1.309072	Down	0.023418	43
Integrin alpha-IIb	−1.218369	Down	0.004697	6	Annexin A5	−1.325927	Down	0.018899	44
Complement C1q subcomponent subunit A	−1.405607	Down	0.000985	7	Platelet endothelial cell adhesion molecule	−1.008523	Down	0.015016	45
Coagulation factor IX	1.349174	Up	0.003383	8	Integrin alpha-6	−1.355566	Down	0.007660	46
Hemoglobin subunit alpha	1.623455	Up	0.022906	9	Protein TFG	−1.209867	Down	0.016495	47
Integrin beta-3	−1.497658	Down	0.003591	10	CD63 antigen	−1.261780	Down	0.022800	48
Properdin	−1.580598	Down	0.029967	11	Histone H3.1	−1.831838	Down	0.015954	49
Angiopoietin-1	−1.054527	Down	0.002045	12	Myc target protein 1	−1.022142	Down	0.019219	50
Serum amyloid P-component	−3.631594	Down	0.000011	13	Amyloid beta A4 protein	−1.187662	Down	0.000046	51
Hemoglobin subunit delta	2.021220	Up	0.018006	14	Flotillin-2	−1.410632	Down	0.001637	52
Complement C1q subcomponent subunit B	−1.269872	Down	0.001464	15	Hemoglobin subunit gamma-1	1.278896	Up	0.015672	53
Disintegrin and metalloproteinase domain-containing protein 10	−1.016039	Down	0.030838	16	Pituitary tumor-transforming gene 1 protein-interacting protein	−1.713387	Down	0.010126	54
Complement C1q subcomponent subunit C	−1.414312	Down	0.003552	17	Syntaxin-7	−1.236001	Down	0.008222	55
Annexin A11	−1.360513	Down	0.016352	18	Serglycin	−2.569884	Down	0.000172	56
Tetraspanin (Fragment)	−1.840424	Down	0.004670	19	Platelet glycoprotein Ib beta chain	−1.602377	Down	0.011227	57
Annexin A7	−1.321818	Down	0.018734	20	Vitamin K-dependent protein C	−2.058997	Down	0.043825	58
Platelet glycoprotein 4	−1.134165	Down	0.020730	21	LIM and senescent cell antigen-like-containing domain protein 1	−1.214167	Down	0.003280	59
Alpha-2-HS-glycoprotein	−1.051564	Down	0.030270	22	Integrin alpha-2	−1.097690	Down	0.003052	60
Ficolin-1	−1.114984	Down	0.022865	23	Tetraspanin-33	−1.713974	Down	0.027229	61
Tetraspanin-14	−1.272294	Down	0.019024	24	Choline transporter-like protein 2	−1.167148	Down	0.030516	62
Integrin beta-1	−1.282317	Down	0.012621	25	Ras-related protein Rap-2b	−1.189959	Down	0.015523	63
Annexin A4	−1.546716	Down	0.006920	26	Biglycan	−1.375279	Down	0.001012	64
Sorcin	−1.109088	Down	0.029105	27	Histone H1.5	−2.099122	Down	0.011138	65
Fermitin family homolog 3	−1.166486	Down	0.002711	28	High affinity immunoglobulin epsilon receptor subunit gamma	−1.211342	Down	0.004402	66
Protein tweety homolog 3	−1.267064	Down	0.014102	29	Junctional adhesion molecule A	−1.158709	Down	0.002309	67
Histone H4	−1.791812	Down	0.022412	30	Copine-1	−1.101457	Down	0.022570	68
Ras-related protein Rap-1b-like protein	−1.587371	Down	0.001839	31	Decorin	−1.596501	Down	0.000112	69
Secreted phosphoprotein 24	−1.041585	Down	0.000366	32	Annexin A1	−1.141563	Down	0.032178	70
Immunoglobulin kappa variable 6D-21	1.103403	Up	0.011645	33	Thymosin beta-4	−1.685965	Down	0.008011	71
Ubiquitin-40S ribosomal protein S27a	−1.090804	Down	0.010559	34	*P*-selectin	−1.393062	Down	0.000135	72
Pleckstrin	−1.253374	Down	0.002195	35	Lysosome-associated membrane glycoprotein 1	−1.546113	Down	0.018872	73
Platelet factor 4	−2.280225	Down	0.000085	36	Alpha-parvin	−1.275746	Down	0.013671	74
Platelet basic protein	−1.250551	Down	0.018861	37	Metalloendopeptidase	−1.662708	Down	0.000045	75
Histone H2B	−1.548494	Down	0.017000	38	Glycosyl-phosphatidylinositol-anchored molecule-like protein	−2.019729	Down	0.000002	76

*FC, fold-change; P, *p*-value.*

### Bioinformatics Prediction Reveals the Role of Exosome Proteins in Epilepsy

**FIGURE 3:**
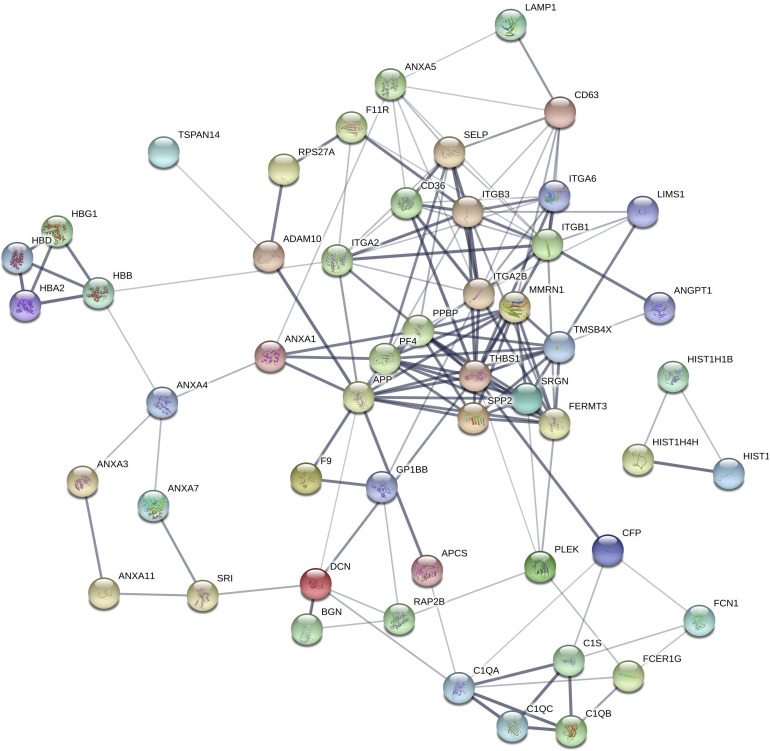
Differential protein interaction network. Each node in the figure represents a protein; each line represents an interaction between proteins, and the width of the line indicates the combined score of the interaction between proteins (0.4–1). The wider a line is, the higher the score, and the narrower a line is, the lower the score; 0.15 indicates a low score, 0.4 indicates a medium score, 0.7 indicates a high score, and 0.9 indicates a very height score. This study selected the default-score minimum value as the medium score (0.4).

To assess the potential biological function of proteins differentially expressed in epilepsy, Gene Ontology (GO) annotation and Kyoto Encyclopedia of Genes and Genomes (KEGG) pathway enrichment analysis were performed. The main GO terms for predicting target genes of differentially expressed proteins mainly include biological process, cellular component and molecular function. Gene enrichment analysis revealed that most of the predicted target genes are involved in cell adhesion, synaptic transmission, signal transduction, cell adhesion and other functions. KEGG pathway analysis revealed that the predicted target genes are involved in biological pathways, including axon guidance, cancer pathway, actin cytoskeleton regulation, focal adhesion, calcium signaling pathway, MAPK signaling pathway and PI3K-Akt signaling pathway. Protein-based networks (Figure 3) were generated based on GO and KEGG prediction data. The results indicate that differentially expressed exosomal proteins may be involved in multiple physiological processes associate with epilepsy.

### Verification That F9 and TSP-1 in Serum Exosomes Can Be Used as Diagnostic Biomarkers

**FIGURE 4:**
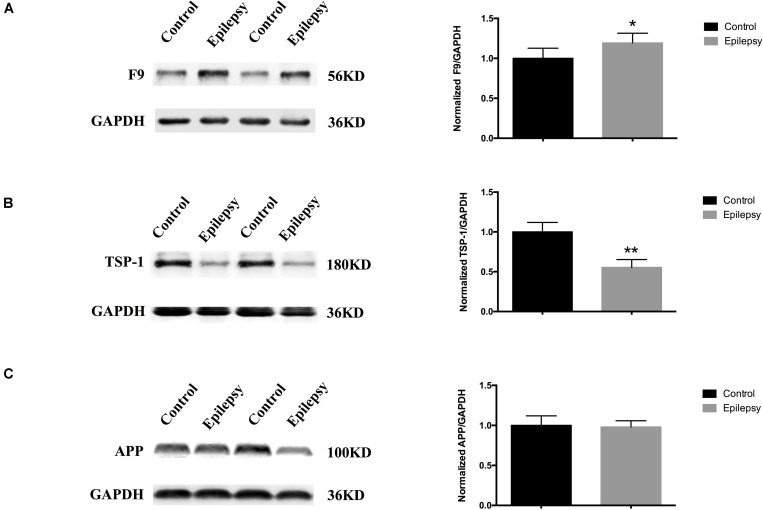
Expression of F9, TSP-1 and APP in serum exosomes from patients with epilepsy. Western blots demonstrated that **(A)** exosomal F9 protein was expressed at higher levels in serum from epilepsy patients than healthy controls (*n* = 25 in each group, ^∗^*P* < 0.05); **(B)** exosomal TSP-1 protein was expressed at lower levels in serum from epilepsy patients than healthy controls (*n* = 25 in each group, ^∗∗^*P* < 0.01); and **(C)** exosomal APP protein was not significantly different in serum from epilepsy patients than healthy controls (*n* = 25 in each group). Mean ± SEM Student’s *t*-test was performed.

**FIGURE 5:**
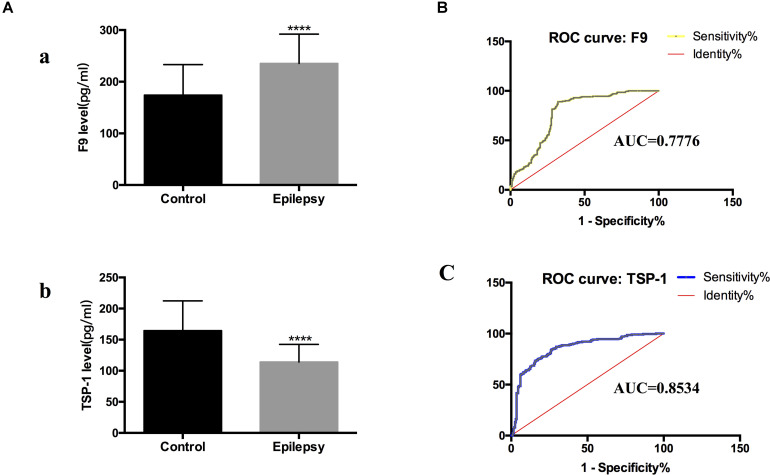
F9 and TSP-1 were verified by ELISA in expanded sample sizes. ROC curves for exosomal proteins that are significantly different in epilepsy patients compared to healthy controls. **(A)** ELISA shows concentrations of F9 **(a)** and TSP-1 **(b)** in serum exosomes in different groups. Mann-Whitney U test was performed, *n* = 200 for each group, ^****^P < 0.0001. **(B,C)** ROC curves for exosomal F9 and TSP-1 were used to discriminate epilepsy patients and healthy controls, and the area under the ROC curve (AUC) was used to evaluate the level of discrimination.

We narrowed down the range of proteins of interest based on quantitative results from proteomics (Table 3) and bioinformatics analysis while paying more attention to proteins with the most significant changes in expression. Three proteins were initially selected: coagulation factor IX (F9), thrombospondin-1 (TSP-1, THBS1), and amyloid-beta precursor protein (APP). The expression levels of these three proteins were verified in serum exosomes of 25 pairs of epilepsy patients and healthy controls (Figure 4) by Western blots. The statistical results showed that compared with that in the healthy controls, the expression of F9 was increased (^∗^*P* < 0.05, Figure 4A) and the expression of TSP-1 was significantly decreased (^∗∗^*P* < 0.01, Figure 4B) in epilepsy; there was no significant difference in APP (Figure 4C). Based on the statistical results, we expanded the sample size (200 pairs of epilepsy patients and healthy controls) to further verify the differential expression of F9 and TSP-1 in serum exosomes by ELISA. The results showed that the expression of F9 in serum exosomes from epilepsy patients was higher than that in serum exosomes from healthy controls (^****^P < 0.0001, Figure 5Aa) and that the expression of TSP-1 in serum exosomes from epilepsy patients was lower than that in serum exosomes from healthy controls (^****^P < 0.0001, Figure 5Ab).

### Evaluation of the Value of F9 and TSP-1 as Potential Diagnostic Markers

To investigate the diagnostic value of exosomal proteins, we used a logistic regression model to assess their differential expression levels between healthy controls and epilepsy patients and to perform receiver operating characteristic (ROC) curve analysis. ROC curves were used to confirm the specificity and sensitivity of F9 and TSP-1 expression in the diagnosis of epilepsy.

Comparing the epilepsy patients with the healthy controls, the capacity of F9 and TSP-1 expression to predict epilepsy yielded an area under the curve (AUC) of 0.7776 (95% CI, 0.7306–0.8246) and 0.8534 (95% CI, 0.8152–0.8916), respectively (Figures 5B,C). These data indicate that serum exosomal proteins are reliable biomarkers for the diagnosis of epilepsy.

### Verification of F9 and TSP-1 in Epilepsy Patients and Mouse Brain Tissue Exosomes

**FIGURE 6:**
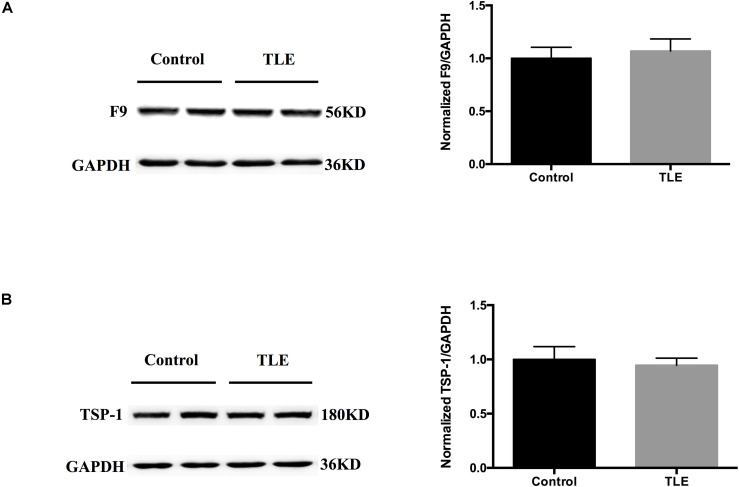
Expression of F9 and TSP-1 in brain tissue exosomes from patients with TLE. **(A,B)** Western blots demonstrated that the levels of exosomal TSP-1 protein and exosomal F9 protein were not significantly different in the temporal lobe tissues of TLE patients (*n* = 4) compared with that of control patients (*n* = 4). Mean ± SEM Student’s *t*-test was performed.

**FIGURE 7:**
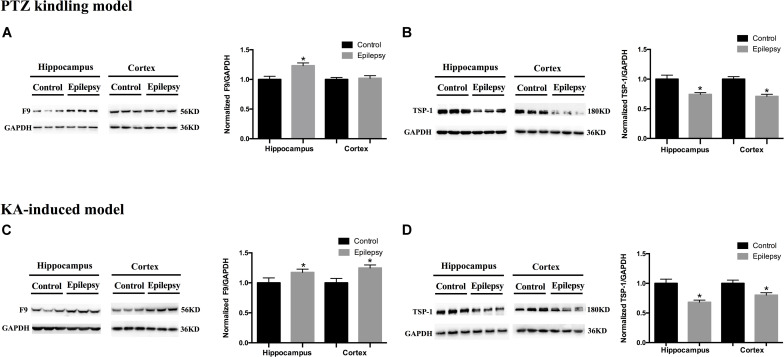
Expression of F9 and TSP-1 in brain tissue exosomes from epileptic mice. **(A)** In the PTZ-kindling epilepsy model exosomal F9 protein was expressed at higher levels in the hippocampus of epileptic mice than in that of control mice (*n* = 5 in each group, ^∗^*P* < 0.05), but there were no significant differences in the levels in the cortex. **(C)** In the KA-induced epilepsy model exosomal F9 protein was expressed at higher levels in the cortex and hippocampus of epileptic mice than in those of control mice (*n* = 5 in each group, ^∗^*P* < 0.05). In the PTZ-kindling epilepsy model **(B)** and the KA-induced epilepsy model **(D)**, exosomal TSP-1 protein was expressed at lower levels in the cortex and hippocampus of epileptic mice than in those of control mice (*n* = 5 in each group, ^∗^*P* < 0.05). Mean ± SEM Student’s *t*-test was performed.

We validated the expression levels of F9 and TSP-1 in human brain tissue exosomes. The differences in these two indicators were not statistically significant (Figures 6A,B). In two mouse models of chronic epilepsy, F9 and TSP-1 were verified in the hippocampus and cortex of mouse tissue exosomes by Western blot analysis (Figure 7). The results showed that TSP-1 was decreased in both the hippocampus and cortex in the two models of epilepsy (^∗^*P* < 0.05, Figures 7B,D); F9 was increased in the hippocampus and cortex in the KA-induced model (^∗^*P* < 0.05, Figure 7C) and the hippocampus in the PTZ-kindling model (^∗^*P* < 0.05, Figure 7A), but the difference was not statistically significant in the cortex of the PTZ-kindling model (Figure 7A).

## Discussion

Exosomes have received increasing attention in the medical community due to their outstanding transmission ability and excellent stability. Advanced and widely used proteomics technology provides an objective basis for the early diagnosis, treatment and prognosis of epilepsy. Some progress has been made in the study of exosomes as biomarkers for neurological diseases. Neural exosomes are diagnostic biomarkers for acute brain injury (Goetzl et al., 2017). Serum exosome SNAP-25 is a potential biomarker of Alzheimer’s disease (Agliardi et al., 2019).

This is the first time that exosomal proteins have been used to study epilepsy. Of particular note in this study was whether exosomes were extracted. We narrowed the range of proteins of interest based on quantitative results from proteomics and bioinformatics analysis while paying more attention to proteins with the most significant changes in expression. Three proteins were initially identified: F9, TSP-1 and APP. We verified the expression levels of these three proteins in serum exosomes, and the results showed that only F9 and TSP-1 were significantly different, which was consistent with the results of TMT proteomic analysis. Based on the statistical results, we verified the expression of F9 and TSP-1 in the serum exosomes of 200 pairs of epilepsy patients and healthy controls. The results showed that the expression of F9 in serum exosomes of epilepsy patients was higher than that in serum exosomes of healthy controls. The expression of TSP-1 in serum exosomes from epilepsy patients was lower than that in serum exosomes from healthy controls. Epilepsy is a slowly progressive brain disease. At present, research on human brain tissue samples is limited to cortical tissues from postoperative epilepsy patients, and the tissue samples that we can obtain and use for verification are postoperative cortical tissues from TLE patients. To further validate and explore the relationship between exosomes and epilepsy, we further validated F9 and TSP-1 in human brain tissue exosomes. The results showed that the differences in these two indicators were not statistically significant. These results may be due to the following factors: (1) The amount of brain tissue we acquired was limited. (2) Brain tissue was derived from TLE patients who took multiple AEDs, but F9 and TSP-1 were screened from newly diagnosed epilepsy patients (not taking any AEDs). (3) The brain tissue resected from TLE patients may have included not only epilepsy lesions but also normal brain tissue surrounding the lesion. Notably, TBI can disrupt the blood-brain barrier and induce coagulopathy (Zhang et al., 2018). Coagulopathy refers to both hypocoagulopathy associated with prolonged bleeding and hemorrhagic progression (Laroche et al., 2012) and hypercoagulopathy with an increased prothrombotic tendency (Sun et al., 2011; Chen et al., 2013), which occur often simultaneously after TBI. Interestingly, both TSP-1 and F9 function as bridging molecules in hemostasis (Hasan et al., 2018; Seif et al., 2018). TSP-1 has been considered a new target for therapeutic development for TBI due to its multiple functions in the neurovascular unit (Chodobski et al., 2011; Lok et al., 2015). In univariate analysis, abnormalities of F9 were significantly associated with mortality after TBI (Kunitake et al., 2017). This may be an important explanation for the lack of differences in both TSP-1 and F9 between TLE and TBI brain tissues, which needs further research.

The hippocampus is the most widely studied brain region in human and experimental epilepsy (Thom, 2014), and we could not perform an equivalent comparison of hippocampal F9 and TSP-1 expression between patients with TLE and controls for practical and ethical reasons. Therefore, we chose two classic chronic epilepsy models to further verify F9 and TSP-1. At the same time, the potential influences of AEDs on F9 and TSP-1 in the TLE brain tissue used for validation were also excluded. In fact, behavioral and molecular events associated with PTZ-kindling and KA-induced epilepsy in mice largely reproduce the corresponding events in human TLE (Ben-Ari, 2001; Wilczynski et al., 2008). The PTZ-kindling model has the advantages of progressive pathological response and epileptic activity (da Silva et al., 1998; Xu et al., 2017). Hippocampal injection of KA produces strong excitatory effects, resulting in excessive excitation and subsequently inducing epileptic seizures (Scerrati et al., 1986). The animal studies provided direct evidence, as TSP-1 expression was lower in the cortex and hippocampus of epileptic mice in the two animal models. In the KA-induced epilepsy model, F9 expression was higher in both the cortex and hippocampus of epileptic mice. In the PTZ-kindling model, F9 expression was higher in the hippocampus but not in the cortex of epileptic mice. This non-significant difference may be due to the insufficient sample size and the epileptic focus in the hippocampus. Furthermore, the diagnostic sensitivity of F9 is indeed not as good as that of TSP-1 from the AUC results, which may be another explanation. Notably, due to technical and sample limitations, protein expression was not examined at the transcriptional level by verifying the expression of associated mRNAs. We verified F9 and TSP-1 at only the protein level, which may have overlooked processes involving related genes at the transcriptional and translational levels; this will be the focus of future research.

TSP-1 is an adhesion glycoprotein that mediates cell-cell and cell-matrix interactions and regulates basic cellular biological processes. In the central nervous system, TSP-1 secreted by astrocytes promotes synapse development, neuronal migration and axonal growth (Christopherson et al., 2005). The interaction of TSP-1 with the 2d-1 subunit stimulates the formation of excitatory synapses, and this observation has led to interest in the potential role of TSP-1 in epilepsy (Meeren et al., 2002). Mendus et al. (2015) found that mice lacking TSP-1 are more sensitive to pentylenetetrazol ignition.

F9 is a vitamin K-dependent plasma protein. It has been reported that coagulation factors can affect the pathophysiology of the central nervous system (CNS). Studies have shown that different doses of thrombin have various effects on the CNS; low doses have beneficial effects on synaptic plasticity (Becker et al., 2014). Different thrombin-sensitive receptors selectively modulate neuronal excitability in different anatomical regions (hippocampus) (Maggio et al., 2013). High doses cause excitotoxicity and apoptosis (Faure et al., 2006; Bushi et al., 2015). These coagulation proteins show unique properties that can also affect synaptic homeostasis, in addition to interfering with coagulation.

Bioinformatics GO analysis showed that F9 and TSP-1 may be involved in many physiological processes of epilepsy. Our research data indicate that F9 and TSP-1 are reliable biomarkers for the diagnosis of epilepsy. Exosomal proteins can be used not only as biomarkers of epilepsy but also to study the mechanism of epilepsy. Epilepsy is a neurological disease. Exosomes contain neuron-specific components. The AMPA receptor subunit GluR2/3 was detected in neuronal exosomes, but conversely, the NMDA receptor subunits NR1 and PSD-95 were undetectable (Faure et al., 2006).

We have discussed the exosomal proteins F9 and TSP-1 as biomarkers for the diagnosis of epilepsy. Moreover, the feasibility of applying exosomes to study the mechanism of epilepsy was briefly analyzed. The study of exosomes in epilepsy is in its infancy. Our study is the first exploration of the use of exosomal proteins for epilepsy. Further exploration of exosomes based on our research will help elucidate the mechanism of exosomes in epilepsy and enable better use of exosomes in clinical applications related to epilepsy.

## Data Availability Statement

The raw data supporting the conclusions of this article will be made available by the authors upon appropriate request.

## Ethics Statement

The study protocol related to human subjects was performed in accordance with the Declaration of Helsinki and approved by the Ethics Committee of the First Affiliated Hospital of Chongqing Medical University. The patients/participants provided their written informed consent to participate in this study. All animal studies were approved by the Ethics Committee of the Chongqing Medical University and were conducted in accordance with the principles outlined in the Animal Research: Reporting In Vivo Experiments (ARRIVE) guidelines.

## Author Contributions

FX, XW, and XT conceived the project and designed the experiments. ZL, YixG, RZ, MW, YiG, YC, JM, FX, and XT performed the experiments. ZL and FX analyzed the data. ZL, XW, and XT wrote the manuscript. All authors revised and approved the final version of the manuscript.

## Conflict of Interest

The authors declare that the research was conducted in the absence of any commercial or financial relationships that could be construed as a potential conflict of interest.
